# Metabolic derangements of skeletal muscle from a murine model of glioma cachexia

**DOI:** 10.1186/s13395-018-0188-4

**Published:** 2019-01-11

**Authors:** Pengfei Cui, Wei Shao, Caihua Huang, Chang-Jer Wu, Bin Jiang, Donghai Lin

**Affiliations:** 1 Department of Chemical Biology, College of Chemistry and Chemical Engineering, Xiamen University, 422 Siming South Road, Xiamen, 361005 China; 2 Department of Pathology, Affiliated Chenggong Hospital of Xiamen University, Xiamen, China; 3 Department of Physical Education, Xiamen University of Technology, 600 Ligong Road, Jimei District, Xiamen, 361024 China; 4 Department of Food Science, National Taiwan Ocean University, Keelung, Taiwan; 5 State Key Laboratory of Cellular Stress Biology, School of Life Sciences, Xiamen University, Xiamen, China

**Keywords:** Glioma cachexia, Malignant grades, Animal model, Muscle atrophy, Metabolic derangements, Skeletal muscle metabolism

## Abstract

**Background:**

Cachexia is a complex metabolic disorder and muscle atrophy syndrome, impacting 80% patients with advanced cancers. Malignant glioma is considered to be one of the deadliest human cancers, accounting for about 60% of all primary brain tumors. However, cachexia symptoms induced by glioma have received little attention. This work aims to explore skeletal muscle atrophy in orthotopic glioma murine models.

**Methods:**

BALB/c nude mice were orthotopicly implanted with normal glial (HEB) and glioma (WHO II CHG5 and WHO IV U87) cells. Cachexia symptoms of mice were depicted by phenotypic, histopathologic, physiological, and biochemical analyses. Muscle atrophy-related proteins were examined by western blot, and the involved signaling pathways were analyzed. NMR-based metabolomic analysis was applied to profile metabolic derangements in the skeletal muscle, including multivariate statistical analysis, characteristic metabolite identification, and metabolic pathway analysis.

**Results:**

Compared with controls, mice implanted with glioma cells exhibit typical cachexia symptoms, indicating a high correlation with the malignant grades of glioma. U87 mice develop cachexia much earlier and more severe than CHG5 mice. The glioma-bearing mice showed significantly decreased skeletal muscle mass and strength, which were associated with suppressed AKT, activated AMPK, FOXO, Atrogin1, and LC3. Interestingly, expressions of MuRF1, MyoD1, and eIF3f were not significantly changed. Consistently, metabolomic analyses elucidate pronounced metabolic derangements in cachectic gastrocnemius relative to controls. Glucose, glycerol, and 3-hydroxybutyrate were remarkably downregulated, whereas glutamate, arginine, leucine, and isoleucine were upregulated in cachectic gastrocnemius. Furthermore, U87 mice showed more characteristic metabolites and more disturbed metabolic pathways including glucose and lipid metabolism, protein catabolism, anabolism, and citric acid cycle anaplerotic.

**Conclusions:**

This study demonstrates for the first time that the orthotopic glioma murine model developed here exhibits high fidelity of cachexia manifestations in two malignant grades of glioma. Signaling pathway analysis in combination with metabolomic analysis provides significant insights into the complex pathophysiology of glioma cachexia and expands understanding of the molecular mechanisms underlying muscle atrophy.

**Electronic supplementary material:**

The online version of this article (10.1186/s13395-018-0188-4) contains supplementary material, which is available to authorized users.

## Background

Cancer cachexia is a devastating and multifactorial syndrome characterized by abnormal metabolism, body weight loss, depletion of skeletal muscle mass, and anorexia [1]. It is most commonly observed in patients with advanced cancers and accounts for approximately 20% of cancer deaths [2]. Body weight loss mostly resulting from muscle atrophy is considered the main hallmark of cancer cachexia [3]. Loss of skeletal muscle makes routine activities difficult and leads a sensation of fatigue, along with the significant impairment of quality of life and poor response to therapy [4]. Fatigue and decreased muscle strength were also proposed as diagnostic criteria for cachexia and extensively used to monitor anti-cachexia treatments [5, 6]. Based on the known molecular mechanisms underlying muscle atrophy during cancer cachexia, skeletal muscle seems to be an ideal target of treatment with cachexia [7]. Research has shown that the prevention of muscle atrophy not only reverses the symptoms of cachexia but also dramatically prolongs survival, without influencing tumor growth [8]. Therefore, mechanistic understanding of cachexia-associated skeletal muscle atrophy is a topic of great interest.

Primary brain tumors, especially glioblastoma, are among the most aggressive human tumors with high mortality rates. According to the WHO standards, gliomas are graded from I to IV, and clinical outcome varies with the tumor grade [9]. WHO III–IV gliomas are designated as malignant or high grade gliomas which can evolve from WHO I–II gliomas, accounting for about 60% of all primary central nervous system (CNS) tumors [10]. Rodent glioma models have been used in preclinical glioma researches for over 30 years; however, no studies have been reported to exploit glioma-associated cachexia. Early in 1988, Griffith et al. have reported anorexia and body weight loss as specific signs of cachexia in glioma patients [11]. Since then the cachexia-associated symptoms of glioma have received little attention. In addition, fatigue, as an another important symptom of cachexia, is among the most common and most severe symptoms in primary brain tumor patients, with an estimated prevalence between 25 and 90% throughout the illness trajectory [12]. Specifically, the prevalence of fatigue in glioma patients varies from 39 to 77% [13]. It has been previously demonstrated that skeletal muscle atrophy is the most important phenotypic feature of cancer cachexia and the principle cause of fatigue [5, 7]. However, systematic studies on cachexia symptoms and skeletal muscle atrophy induced by glioma have rarely been reported. Furthermore, inspiring outcomes have been achieved through both appropriately treating with cachexia and effectively preventing muscle atrophy in non-CNS cancers [14], which prompt us to explore the cachexia symptoms in CNS tumors. Expectedly, such studies will provide novel insights and meaningful therapeutic advances to improve clinical outcomes, quality of life, and survival for cachexia patients with glioma or brain tumors.

Recently, metabolomics has acquired a great interest because the metabolites are the end points of biological processes, and therefore, it is assumed that they can reflect all the variables that contribute to the phenotype [15]. Metabolomic analyses of the small-molecule metabolite profiles have been used to obtain better understandings of cancer cachexia [16–18]. Although previous studies have found that blood hyperlipidemia and hypoglycemia are associated with cancer cachexia [19, 20], the specificity of these markers has been challenged, and little is known about the impact of muscle atrophy on muscle functions and metabolic properties [16, 21, 22]. In addition, the metabolic signatures and molecular mechanisms of non-CNS cancer cachexia are not mostly applicable for brain CNS tumors. Unlike non-CNS solid tumors, brain tumors especially glioma can widely invade normal brain tissues, but rarely metastasize outside the CNS [23]. As well known, metastases act usually as a most frequent complication of cancer in the non-CNS, such as pancreatic, gastric, and lung cancers, a greater burden of metastases, and a higher risk of cachexia [3]. Thus, it is conceivable that the metabolic mechanisms of CNS cancer cachexia would be distinctly different from those of non-CNS cancer cachexia.

As expected, accurate animal models are essential for addressing glioma cachexia with different malignant grades. Previously, we performed NMR-based metabolomic analysis on five glioma cell lines with different malignant grades (WHO II gliomas: CHG5, SHG44; WHO IV gliomas: U87, U118, U251) and revealed that the cellular metabolic profiles were closely associated with the malignant features of glioma cells [9]. Here, we established a glioma cachexia murine model in which BALB/c nude mice were orthotopicly implanted with normal glial (HEB) and glioma (CHG5 and U87) cell lines. This animal model was employed to mimic clinical manifestations in two malignant grades of glioma, in which we have clearly observed glioma cachexia symptoms based on the definition and criteria of cachexia [1].

In the present study, we elucidated metabolic properties of cachectic gastrocnemius by using nuclear magnetic resonance (NMR)-based metabolomics technique. A combination of signaling pathway analysis and metabolic pathway analysis provides significant insights into the complex pathophysiology of muscle atrophy during glioma cachexia, which would be essential for developing new therapeutics.

## Methods

### Cell cultures

The normal glial cell line HEB was obtained from Beinuo life science Company in Shanghai [24]. Human glioma cell line CHG5 (WHO II) was kindly provided by Professor XW Bian of the Third Military Medical University, China [9]. The human glioma cell line U87 (WHO IV) was obtained from the American Type Culture Collection (ATCC, HTB-14). Murine C2C12 myoblasts were purchased from the China Center for Typical Culture Collection (CCTCC). All the cell lines were maintained in DMEM supplemented with 100 units/ml penicillin, 100 μg/ml streptomycin, and 10% fetal bovine serum (FBS, Hyclone) at 37 °C in a humidified atmosphere of 5% CO_2_. C2C12 myoblasts were cultured in growth medium (DMEM supplemented with 10% fetal bovine serum). At 85% confluence, myoblast differentiation was induced by incubation for 96 h in differentiation medium (DMEM supplemented with 2% horse serum) to form myotubes. The conditioned medium from cultured HEB and glioma cells (cells cultured in medium for 48 h) was collected and centrifuged (450 g, 5 min, 4 °C); the medium was diluted by four times with 2% horse serum before adding to C2C12 myotubes [25]. In this study, all types of cell lines were used within 3–8 generations of culture.

### Animal experiments

All animal studies were performed according to protocols approved by Xiamen University Institutional Animal Care and Use Committee. Six to 8-week-old female BALB/c nude mice were housed in Xiamen University Laboratory Animal Center and maintained in conditions of constant temperature and 12-h light/12-h dark cycles. Mice were injected into their right caudate nucleus with 2 μL of the suspension of 2.0 × 10^5^ of HEB, CHG5, and U87 cells using the method of Calin et al. [26]. The three groups of mice were defined as HEB mice (controls), CHG5 mice, and U87 mice, respectively. Body weights were monitored every 2–3 days. Forelimb grip force was measured using a Grip Strength Meter (YLS-13A, Shandong Academy of Medical Sciences, China). For each mouse, grip strengths were defined as the average of five measurements. Mice were sacrificed on day 26 of the study period based on the criteria of cachexia [1]; the brains and muscles were rapidly dissected, weighted, and frozen in liquid nitrogen and stored at − 80 °C until analyses. Fundus blood was obtained from the retro-orbital plexus under ether anesthesia, and then, serum samples were prepared.

### Histopathology

Right brains and gastrocnemius muscles derived from the three groups of mice were fixed in 4% paraformaldehyde. After dehydration, the biopsies were embedded in wax and stained with hematoxylin and eosin for histopathologic examination by light microscopy. Myofiber cross-sectional areas (CSAs) in the gastrocnemius were quantified based on the H&E image by the ImageJ software (National Institutes of Health, Frederick, MD, USA).

### Western blot

Gastrocnemius was homogenized and solubilized in RIPA lysis buffer with the protease-inhibitor cocktail (Roche). Muscle homogenates were then sonicated for 30 s and centrifuged (13,000*g* for 10 min at 4 °C) to remove the debris. The supernatants were collected, and protein concentrations were determined by BCA Protein Assay Kit (Thermo). Equal amounts of proteins were subjected to SDS-PAGE and transferred to PVDF membranes (GE Healthcare) for immunoblotting analysis. The membranes were blocked in 5% milk in Tris-buffered saline with 0.1% Tween 20 (TBST) and then incubated with primary and secondary antibodies in 5% milk in TBST. Relevant references for primary antibodies are showed in Additional file 1: Table S1. Secondary antibodies included HRP-conjugated anti-rabbit and anti-mouse antibodies (Multi Sciences). Finally, blots were visualized by enhanced chemiluminescence reagents (Amersham Biosciences). GAPDH served as an internal control.

### NMR sample preparation

Aqueous intracellular metabolites were extracted from gastrocnemius for the NMR analysis according to the protocol described previously [27]. Generally, gastrocnemius samples were homogenized after adding ice-cold methanol, chloroform, and water at a volume ratio of 4:4:2.85 to obtain a two-phase extract. Only the upper polar tissue extracts were lyophilized and suspended in 550 μL of NMR buffer (50 mM sodium phosphate buffer, pH 7.4, in D_2_O) using 0.1 mM sodium 3-(trimethylsilyl) propionate-2,2,3,3-d4 (TSP). D_2_O was used for field-frequency lock, and TSP was used to provide the chemical shift reference (δ 0.00). All the samples were vortexed uniformly and then centrifuged (12,000*g* for 10 min at 4 °C). The supernatants were transferred into a 5-mm NMR tubes.

### NMR measurements

All NMR experiments were performed on a Bruker Avance III 850 MHz spectrometer (Bruker BioSpin, Germany) equipped with a TCI cryoprobe at 25 °C. One dimensional (1D) ^1^H spectra were recorded on aqueous extracts of gastrocnemius using the pulse sequence NOESYGPPR1D [RD–G_1_-90°–t–90°–τ_m_–G_2_-90°–ACQ] with water suppression during the relaxation delay and mixing time. RD was the relaxation delay (4 s), *t* was a short delay (4 μs), and *τ*_m_ was the mixing time (10 ms). Pulsed gradients G_1_ and G_2_ were used to improve water suppression quality. A total of 32 transients were collected into 64 K data points using a spectral width of 17 KHz with an acquisition time (ACQ) of 1.88 s. For the purpose of metabolite resonance assignments, two-dimensional (2D) ^1^H-^13^C heteronuclear single quantum coherence (HSQC) spectra were recorded on selected NMR samples. Identification of metabolites was accomplished using the Chenomx NMR Suite software (version 8.2, Chenomx Inc., Canada) based on the 1D ^1^H spectra. Identified metabolites were confirmed by a combination of 2D NMR data and the Human Metabolome Data Base (HMDB), referring to the relevant published reference [28].

### NMR data processing

NMR spectral data processing was carried out using the MestReNova software (version 9.0, Mestrelab Research S. L, Spain). The free induction delay (FID) signals were processed by applying an exponential function with a line-broadening factor of 0.3 Hz prior to Fourier transformation, followed by manual phasing and baseline correction. The NMR spectra of aqueous extracts were referenced to the methyl group of TSP (δ 0.00). The spectral regions of *δ* 10.0–0.6 were binned by 0.003 ppm. Regions of water resonance *δ* 5.1–4.7 were removed from the spectra. The remaining peak integrals for each NMR spectrum were normalized by the sum of the peak integrals to compensate for differences in sample concentrations. Relative levels of the identified metabolites were presented by the normalized peak integrals. For relative quantification, the intensity of each metabolite was calculated by using the relative integral of singlet or non-overlapped signals in each NMR spectrum and was represented as mean ± standard deviation (SD).

### Metabolomic analysis

Multivariate statistical analysis was conducted using the SIMCA-P+ software (version 12.0.1, Umetrics, Sweden). Pareto scaling was applied to the normalized NMR data for increasing the importance of low-level metabolites without significant amplification of noise. Then, principal component analysis (PCA) was performed to reveal metabolic separation and show clusters among samples. In addition, partial least-squares discriminant analysis (PLS-DA) was subsequently used to improve the metabolic separation. The PLS-DA model was cross-validated to evaluate the robustness by a response permutation test (RPT) with 200 cycles. The extracted *R*^2^ and *Q*^2^ values reflected the explained variance and predictive capabilities. The reliability of the model was increased with *R*^2^ and *Q*^2^ approaching to 1 [29, 30]. One-way analysis of variance (ANOVA) followed by Tukey’s multiple comparison test were used to perform comparisons of metabolite levels among the three groups of mice. A clustered heatmap plot of relative metabolite levels was applied to visualize significantly changed metabolites in cachectic mice relative to controls. Metabolite set enrichment analysis (MSEA) was conducted to determine distinctly altered metabolic pathways based on differential metabolites identified from the pair-wise comparisons of U87 mice vs. controls and CHG5 mice vs. controls (*P* < 0.05). Both the heatmap and MSEA were implemented by using the MetaboAnalyst 3.0 suite [31].

### General statistical analysis

Experimental results were reported as mean ± SD. For quantitative comparison between two groups, data were analyzed by one-way ANOVA using the GraphPad Prism software (version 6.0, La Jolla, USA). Statistically significances are as follows: *P* > 0.05 (NS), *P* < 0.05 (*), *P* < 0.01 (**), and *P* < 0.001(***).

## Results

### Glioma-bearing mice exhibit cancer cachexia symptoms

**Fig. 1:**
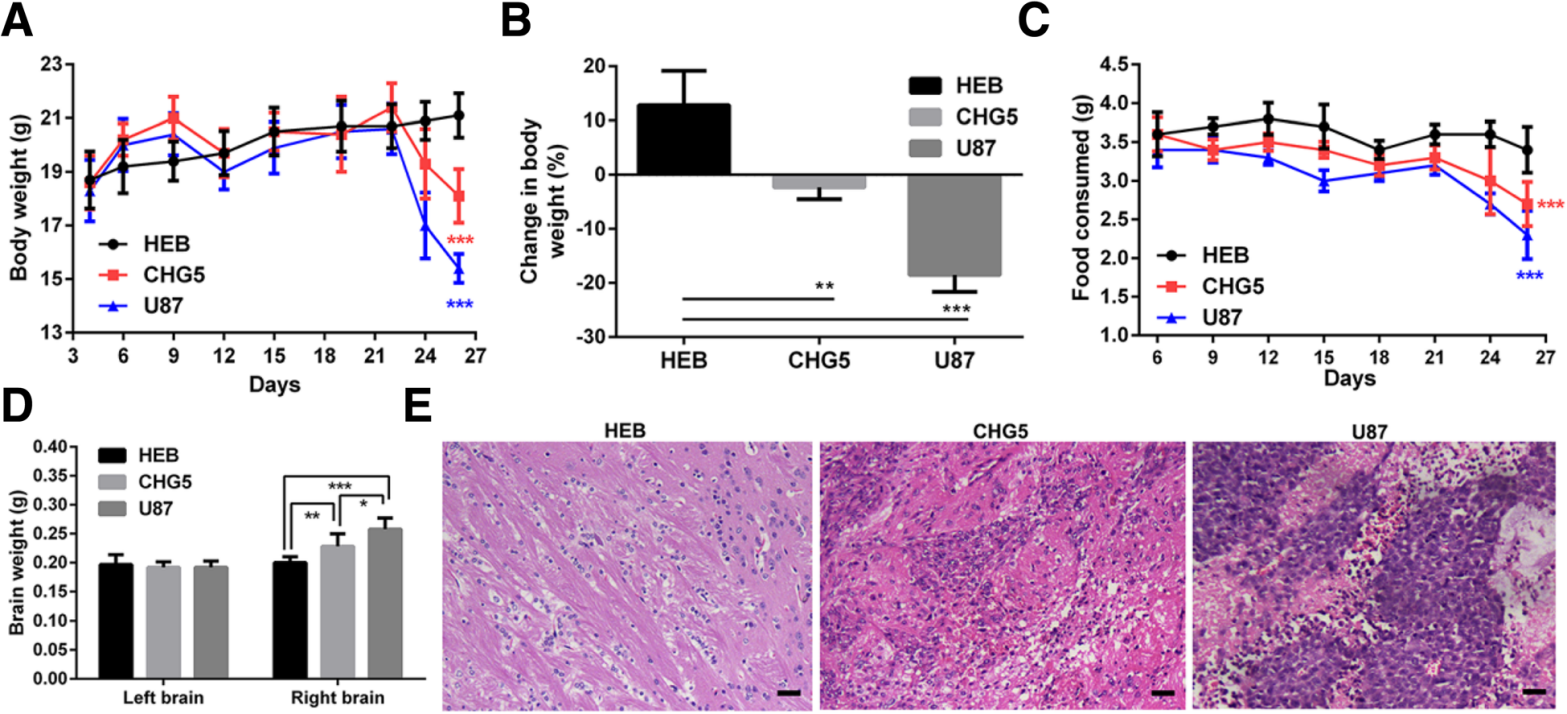
Orthotopic glioma murine model exhibiting evident body weight loss and glioma formation. The model was established by implanting three human cell lines into the right lateral ventricles of the mice. HEB, CHG5, and U87 represent normal glial, low-grade glioma, and high-grade glioma cells, respectively. Mice inoculated with HEB cells served as the control group. **a** Body weights of the mice over the course of the study. **b** Body weight changes in glioma-bearing mice at day 26 of the study period, relative to their initial weights. **c** Average daily food consumption per mouse over the course of the study period. **d** Weights of left and right brains of the mice at day 26 of the study period. **e** Representative micrographs of HE histology of right brains in glioma-bearing mice, relative to controls. Scale bars, 100 μm. Values that were significantly different from the control group were evaluated using one-way ANOVA. Data are presented as mean ± SD (*P < 0 .05; **P < 0.01; ***P < 0.001)

In this study, we successfully established a glioma-associated cachexia murine model. This model was established by implanting three human cell lines into right lateral ventricles of the mice, including HEB, CHG5, and U87. Compared with controls, body weights of CHG5 and U87 mice showed a severe and moderate decrease (*P* < 0.001) (Fig. 1a). Relative to their initial body weights, CHG5 and U87 mice decreased body weights of 2.31% and 18.5% at day 26 of the study period (Fig. 1b). Food consumption data indicated that glioma-bearing mice suffered anorexia particularly obvious in U87 mice, in which average daily diet per mouse continually declined (Fig. 1c). CHG5 and U87 mice displayed 14% and 29% increases in the right brain weights compatible with tumor mass (*P* < 0.01 and *P* < 0.001), respectively. U87 mice suffered a larger tumor burden than CHG5 mice (*P* < 0.05). There was no difference in the left brain weights among the three groups of mice (Fig. 1d). Histological analysis of right brains strengthened these results. Obvious xenograft glioma formations were detected in glioma-bearing mice, while no tumor developed in controls. Right brains of U87 mice sustained more obviously nuclear atypia and mitosis than those of CHG5 mice, corroborating that U87 mice suffered a higher malignant grade xenograft glioma than CHG5 mice (Fig. 1e). At necropsy, no brain edema and tumor metastasis were observed in glioma-bearing mice. Overall, mice implanted orthotopicly with glioma cell lines exhibited obvious cachexia symptoms. Most notably, U87 mice developed cachexia more severely than CHG5 mice.

### Skeletal muscle atrophy in glioma-bearing mice

**Fig. 2:**
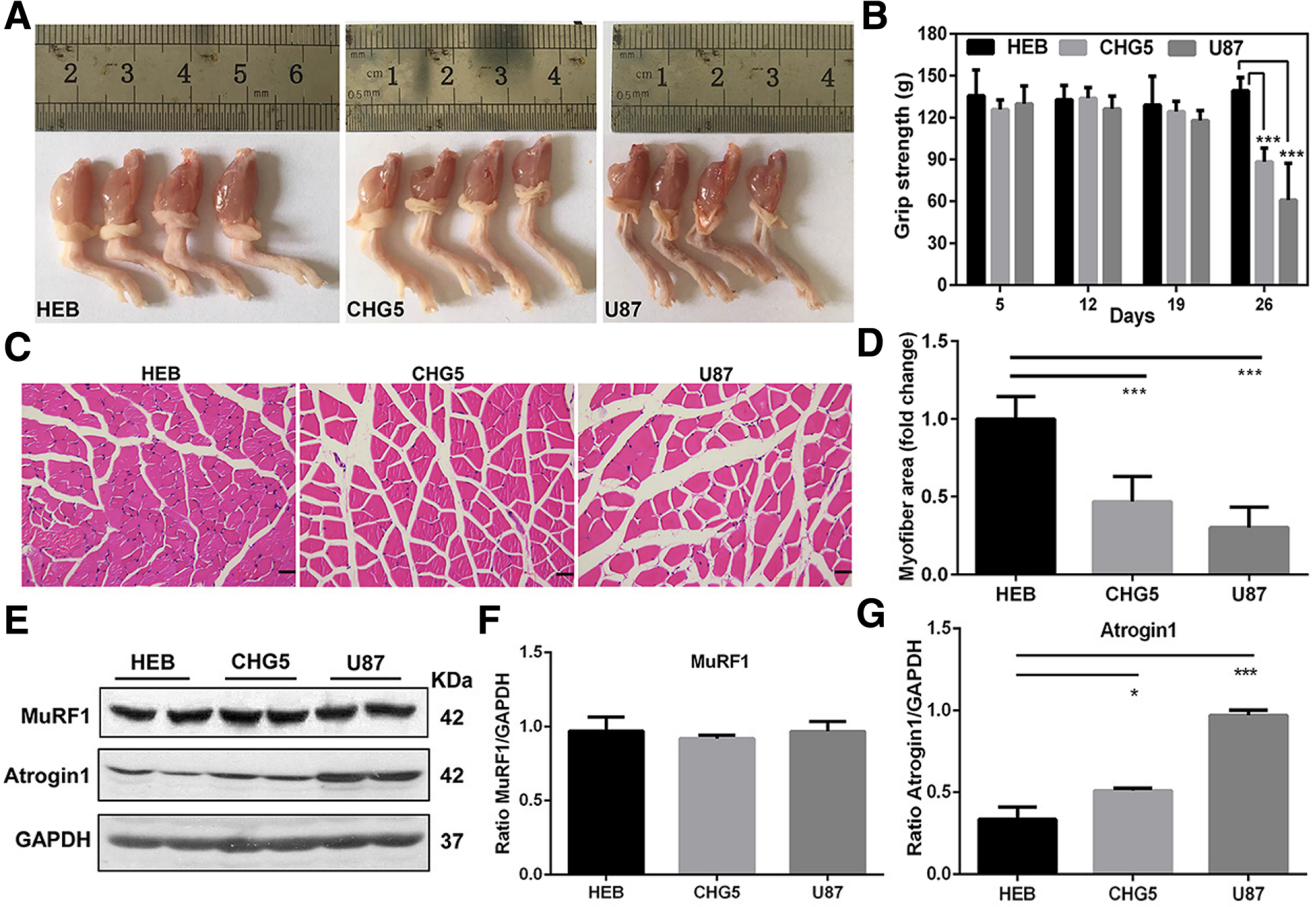
Atrophy and expression of E3 ubiquitin ligases in the gastrocnemius muscle in glioma-bearing mice at the time point after implantation. **a** Macroscopic observation of gastrocnemius muscles in cachectic mice (CHG5 and U87) relative to tumor-free mice (HEB). **b** Grip strengths of forelimbs monitored every 7 days. **c** Representative micrographs of HE histology of gastrocnemius muscles. Scale bars, 20 μm. **d** Quantification of the myofiber cross-sectional areas in glioma-bearing mice relative to HEB mice. **e**–**g** Expressions of two E3 ubiquitin ligases MuRF1 (**f**) and Atrogin1 (**g**) measured by western blot. Values that were significantly different from the control group were evaluated using one-way ANOVA. Data are presented as mean ± SD (*P < 0 .05; **P < 0.01; ***P < 0.001)

Representative images of gastrocnemius showed that the muscles in glioma-bearing mice were significantly smaller than those in controls, and the sizes and weights were decreased with the increasing glioma grade (Fig. 2a). In addition, grip strengths were decreased by 25% and 50% at day 26 of the study period in CHG5 and U87 mice, respectively, relative to controls (*P* < 0.001, Fig. 2b). Histopathologic analysis further indicated that muscle loss of the glioma groups became more severe as the malignant grade increased (Fig. 2c). Myofiber cross-sectional area in the gastrocnemius was significantly smaller in glioma-bearing mice than those in HEB mice (Fig. 2d). Two molecular markers, E3 ubiquitin ligases MuRF1 and Atrogin1, were used to confirm muscle atrophy in glioma-bearing mice. Significantly enhanced expressions of Atrogin1 were characterized in U87 mice (*P* < 0.001), while slightly increased expressions of Atrogin1 were observed in CHG5 mice (*P* < 0.05, Fig. 2e, g). However, the expressions of MuRF1 did not display statistically significant difference among the three groups of mice (Fig. 2e, f). Overall, decreases in muscle mass and strength were evident in glioma cachectic mice.

### Skeletal muscle atrophy-related proteins in glioma-bearing mice

**Fig. 3:**
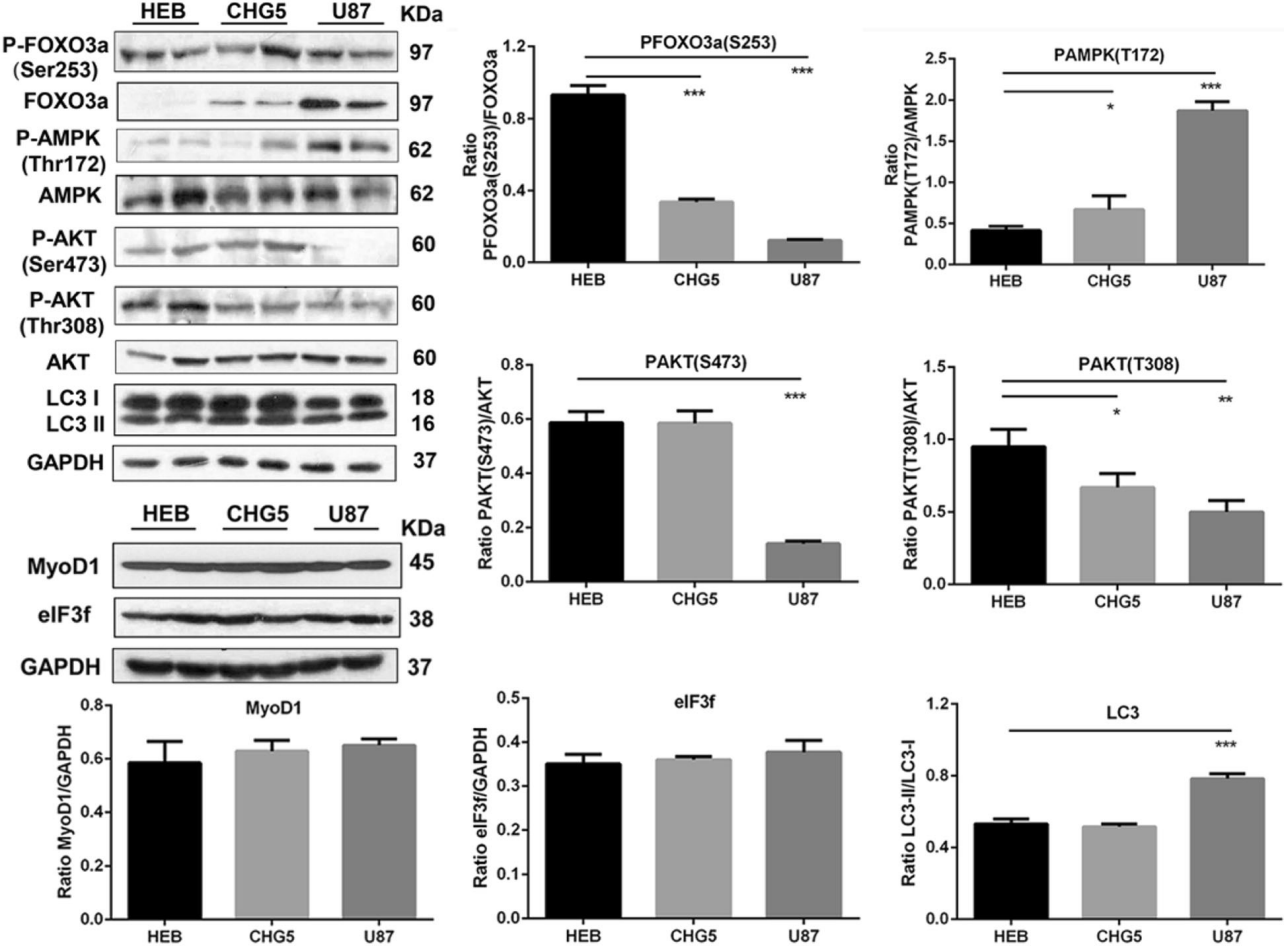
Expressions of skeletal muscle wasting-related proteins in gastrocnemius muscles of mice. Western blot analyses for proteins including FOXO3a, AMPK, AKT, LC3, MyoD1, and eIF3f. Values significantly different from the control group were evaluated using one-way ANOVA. Data are presented as mean ± SD (*P < 0.05; **P < 0.01; ***P < 0.001 vs. the control group)

As described above, the expressions of Atrogin1 were increased in glioma-bearing mice. Also, the microtubule-associated protein 1 light chain 3 (LC3) conversion ratio (LC3-II/LC3-I) was significantly increased in U87 cachectic skeletal muscles. Moreover, we detected a downregulated expression of phosphorylated O-type forkhead FOXO3a, which potentially acted as one of the transcriptional inducers of Atrogin1 and LC3 (*P* < 0.001, Fig. 3). Furthermore, we analyzed the AMP-activated protein kinase (AMPK) and AKT to explore the connection between protein breakdown and protein synthesis, and also analyzed the regulators of FOXO3a (Fig. 3). The analysis of AMPK phosphorylation at Thr 172 exhibited that AMPK activity was markedly increased in cachectic muscles relative to controls (*P* < 0.05 and *P* < 0.001, respectively, Fig. 3). The amounts of phosphorylated AKT at Thr 308 were diminished in cachectic muscles of both CHG5 and U87 mice compared with HEB mice (*P* < 0.05 and *P* < 0.01, respectively, Fig. 3). In contrast, the reduction in AKT phosphorylation at Ser 473 was only observed in U87 mice relative to controls (*P* < 0.001, Fig. 3). Interestingly, some relevant proteins were not significantly changed, including myogenic differentiation 1 (MyoD1) and eukaryotic initiation factor (eIF3f) (Fig. 3). These results indicated that muscle atrophy in glioma cachexia was mediated by suppressed AKT, activated AMPK, FOXO, Atrogin1, and LC3.

### Metabolic derangements in the glioma cachectic gastrocnemius

**Fig. 4:**
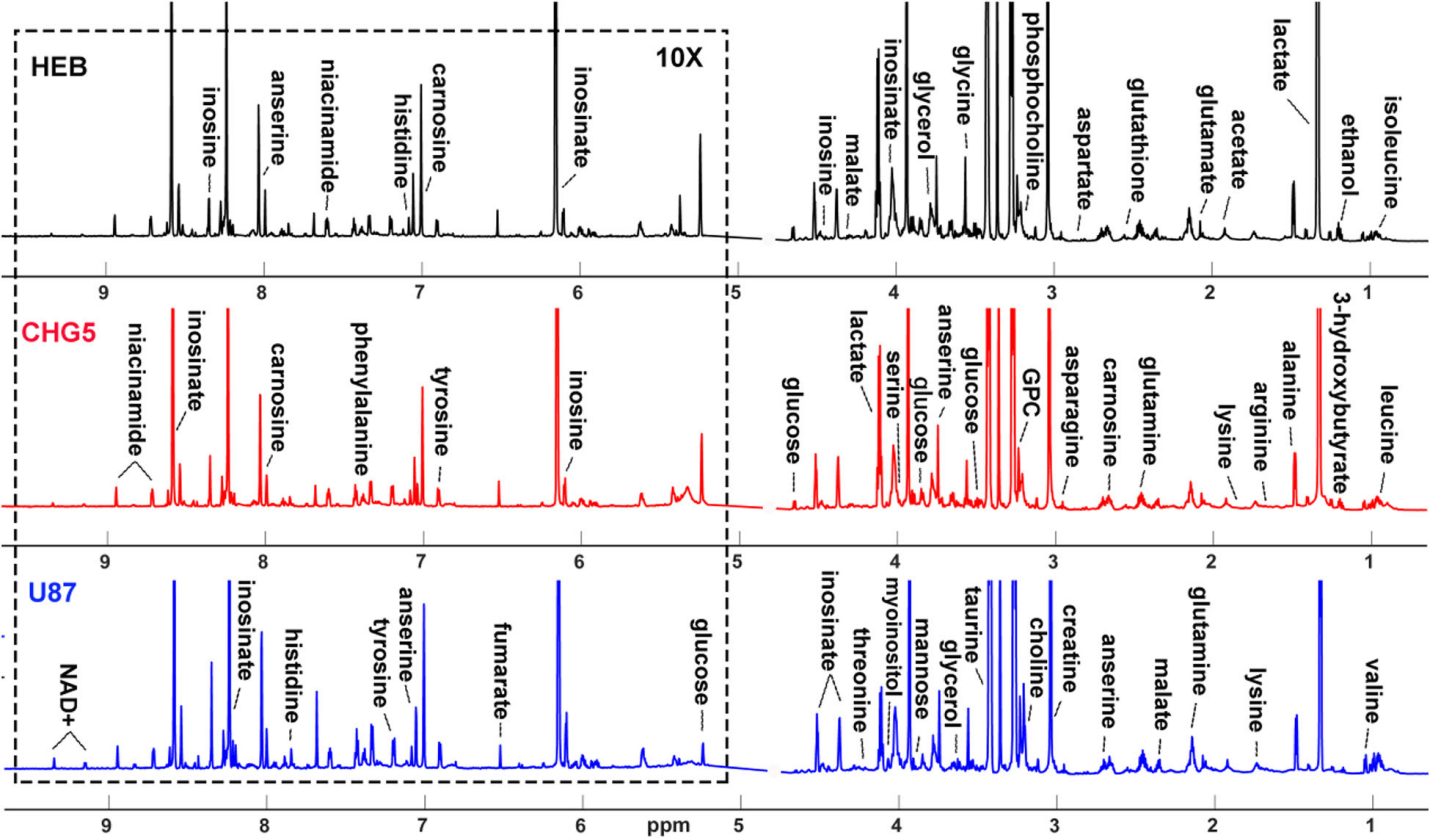
Average 1D ^1^H NOESY spectra of aqueous extracts derived from gastrocnemius muscles of mice. The spectra were recorded on a Bruker Advance III 850 MHz NMR spectrometer at 25 °C. The regions of water resonance were removed from the spectra

**Fig. 5:**
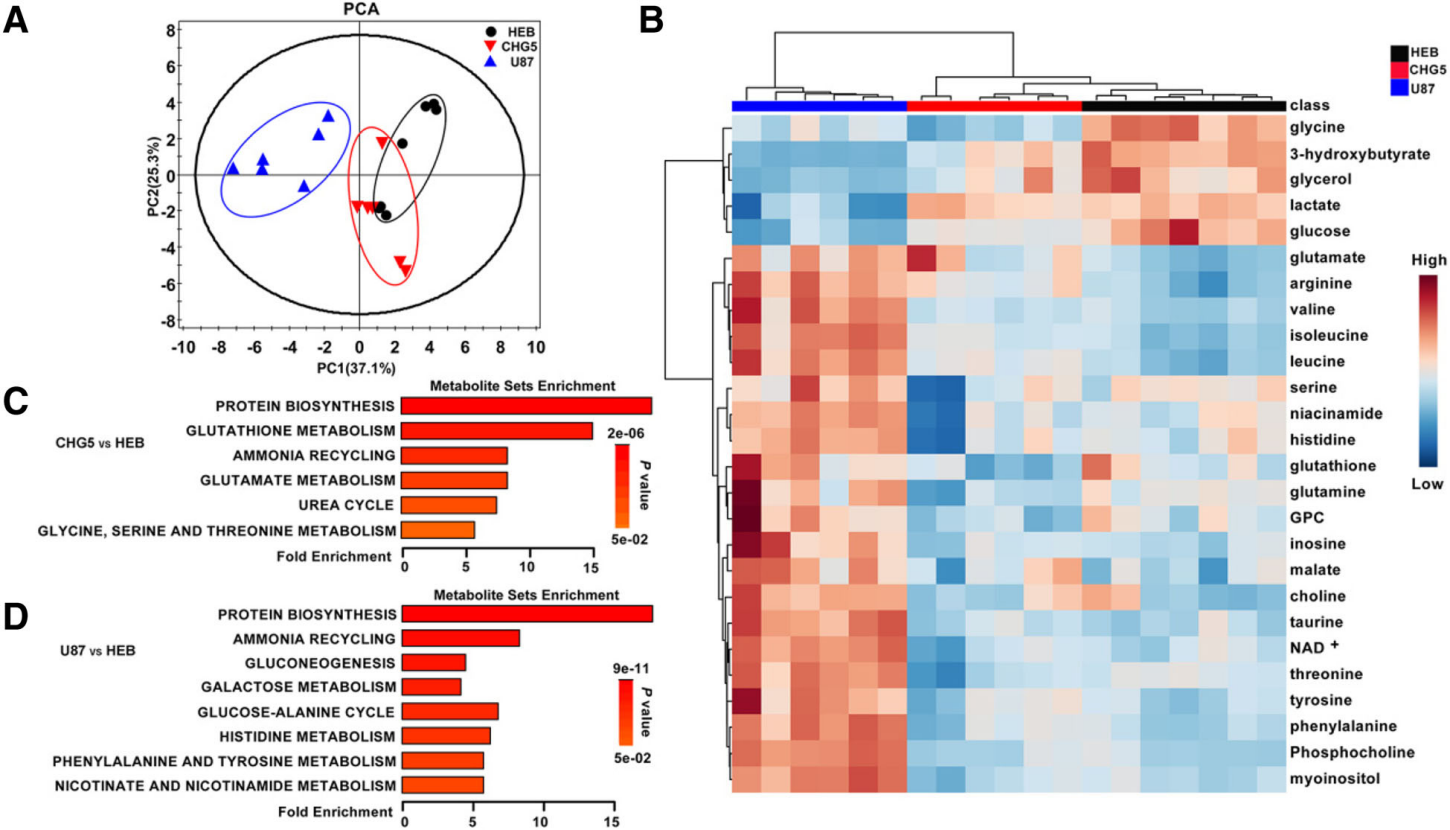
Metabolomic analysis reveals pronounced metabolic disorders in gastrocnemius muscles of glioma-bearing mice compared to controls. **a** PCA scores plot demonstrating distinct metabolic differences among the three groups of mice. The *x*-axis represents the first PC accounting for 37.1% of the total variation. The *y*-axis denotes the second PC accounting for 25.3% of the total variation. Ovals are showed in the panel to highlight metabolic distinctions. **b** Clustered heatmap plot of relative metabolite levels showing metabolic derangements in glioma cachectic mice relative to controls. One-way ANOVA analysis followed by Tukey’s multiple comparison test was used to perform multiple comparisons of metabolite levels among the three groups of mice (*n* = 6–7; *P* < 0.05). **c**, **d** Metabolite set enrichment analyses (MSEA) for identifying significantly altered metabolic pathways (*P* < 0.05) of CHG5 vs. HEB (**c**) and U87 vs. HEB (**d**)

Average 850 MHz 1D ^1^H NMR spectra of aqueous extracts of gastrocnemius muscles are showed in Fig. 4. 2D ^1^H-^13^C HSQC spectra recorded on representative NMR samples were used to confirm the identifications of metabolite resonances, especially the crowded resonance regions in the 1D ^1^H NMR spectra (Additional file 1: Figure S1). To obtain a comprehensive comparison of metabolic profiles among the three groups of mice, we conducted multivariate statistical analysis on the NMR data. PCA scores plot is shown with the first two principal components (PC1 and PC2, Fig. 5a). U87 mice displayed distinctly different metabolic profiles from CHG5 and HEB mice. Furthermore, the PLS-DA scores plots illustrate distinct metabolic separations between HEB and CHG5, HEB and U87, and CHG5 and U87 (Additional file 1: Figure S2A–C). The validation plots of the corresponding RPTs confirm that the three PLS-DA models are valid (Additional file 1: Figure S2D–F).

In summary, both PCA and PLS-DA scores plots demonstrate that the metabolic profiles of CHG5 and U87 groups are clearly distinguished from that of the control group. Based on relative integrals calculated from the 1D NMR spectra of aqueous gastrocnemius extracts, we quantified relative levels of the metabolites (Additional file 1: Table S2). The clustered heatmap plot shows the relative levels of 26 significantly changed differential metabolites in the three groups of mice (*P* < 0.05; Fig. 5b). These changed metabolites were mostly concentrated in carbohydrates, lipids, and amino acids (Additional file 1: Table S2). In cachectic muscles, glucose, glycerol, 3-hydroxybutyrate, and glycine were remarkably downregulated. By contrast, glutamate, arginine, leucine, and isoleucine were upregulated with the elevating malignant grade of glioma. The levels of some metabolites in U87 mice were higher than those in HEB mice, which included taurine, myoinositol, phenylalanine, valine, tyrosine, inosine, histidine, NAD+, malate, choline, phosphocholine, and glycerophosphocholine. However, the level of lactate showed a marked decrease in U87 mice and did not display statistically significant difference between HEB and CHG5 mice.

**Fig. 6:**
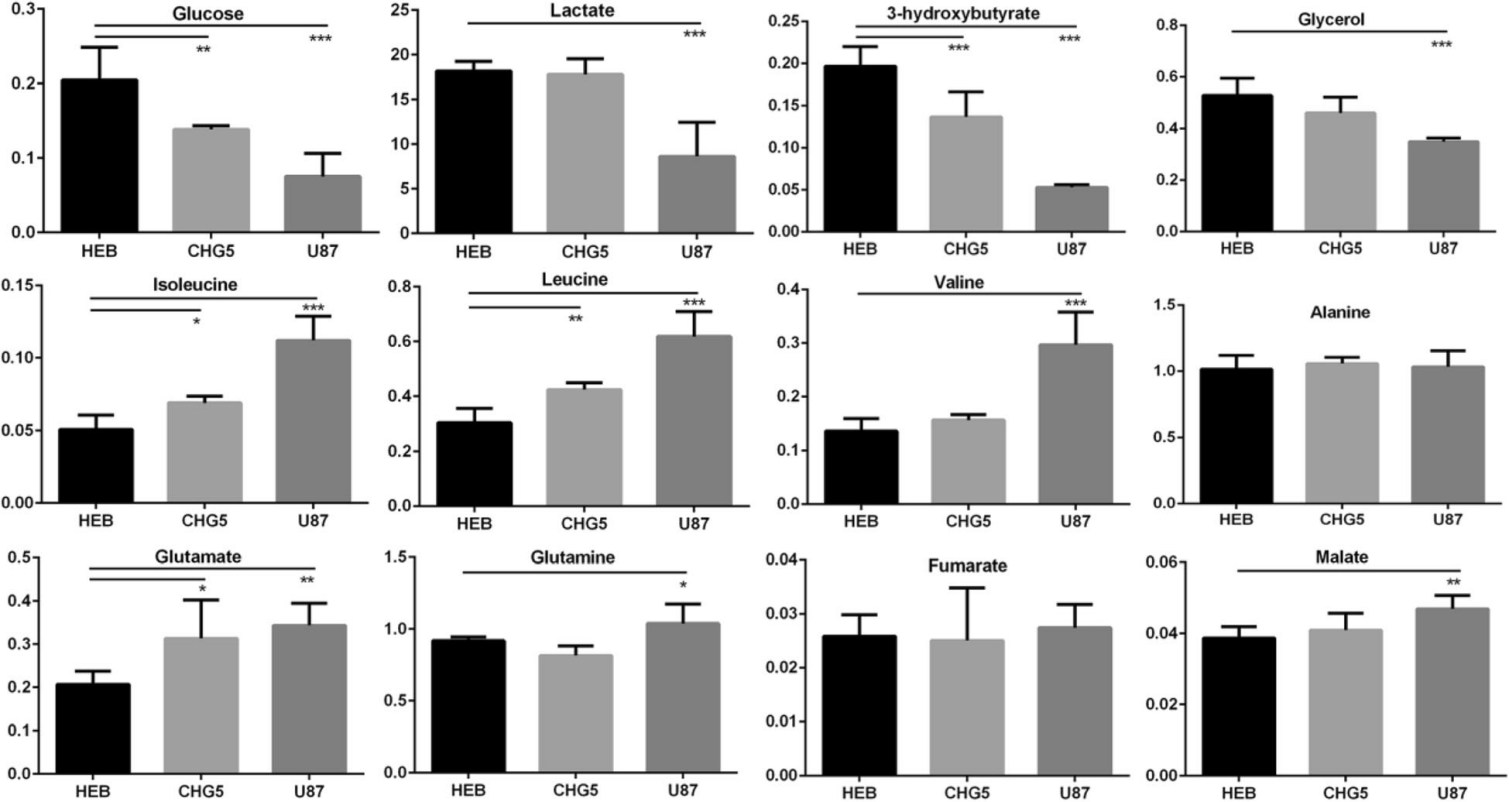
Relative metabolite levels measured from 1D ^1^H NMR spectra of aqueous extracts derived from gastrocnemius muscles of three groups of mice. Values significantly different from the control group were evaluated using one-way ANOVA (*n* = 6–7). Data are presented as means ± SD (*P < 0.05; **P < 0.01; ***P < 0.001 vs. the control group)

Interestingly, U87 mice exhibited enhanced levels of threonine, niacinamide, and glutamine in the gastrocnemius relative to HEB mice, while CHG5 mice displayed declined levels of these metabolites. Compared with controls, CHG5 mice showed the most marked differences for leucine and isoleucine with 1.39 and 1.35 times increases, and also for glucose and 3-hydroxybutyrate with 1.47 and 1.44 times decreases, respectively (Fig. 6). Furthermore, the comparison of U87 mice with controls showed increases of 2.21, 2.17, and 2.03 folds in isoleucine, valine, and leucine and decreases of 3.72-, 2.71-, and 2.11-folds in 3-hydroxybutyrate, glucose, and lactate, respectively (Fig. 6).

Based on the differential metabolites identified from the aqueous gastrocnemius extracts, we performed MESA to identify significantly altered metabolic pathways for glioma-bearing mice compared with controls (*P* < 0.05). We found that protein biosynthesis was the most significantly disturbed metabolism in glioma-bearing mice relative to HEB mice (Fig. 5c, d). Regarding CHG5 mice, six distinctly altered metabolic pathways were identified (Fig. 5c). More significantly, four of these metabolic pathways included glutathione metabolism, glutamate metabolism, urea cycle, and glycine, serine, and threonine metabolism. Regarding U87 mice, eight markedly altered metabolic pathways were identified (Fig. 5d). Notably, six of these metabolic pathways covered gluconeogenesis, galactose metabolism, glucose-alanine cycle, histidine metabolism, phenylalanine and tyrosine metabolism, nicotinate, and nicotinamide metabolism. Taken together, these results revealed that glioma cachexia induced complex metabolic derangements in the gastrocnemius, and the metabolic profiles were closely associated with glioma grades.

## Discussion

Inspiring outcomes have been achieved through treating with cachexia and preventing muscle loss [14]. Such approaches could increase tolerance to anticancer treatments such as chemotherapy and surgery. Conceivably, exploring glioma cachexia will provide novel insights and meaningful therapeutic advances on both glioma and cachexia interventions. In the present study, we established a glioma cachexia murine model by implanting nude mice with the human glioma cell lines CHG5 and U87. The presence of cachexia induced by gliomas was confirmed by the significant body weight loss and decreased muscle weight and strength, the increase of Atrogin1 protein expression in muscle tissues.

**Fig. 7:**
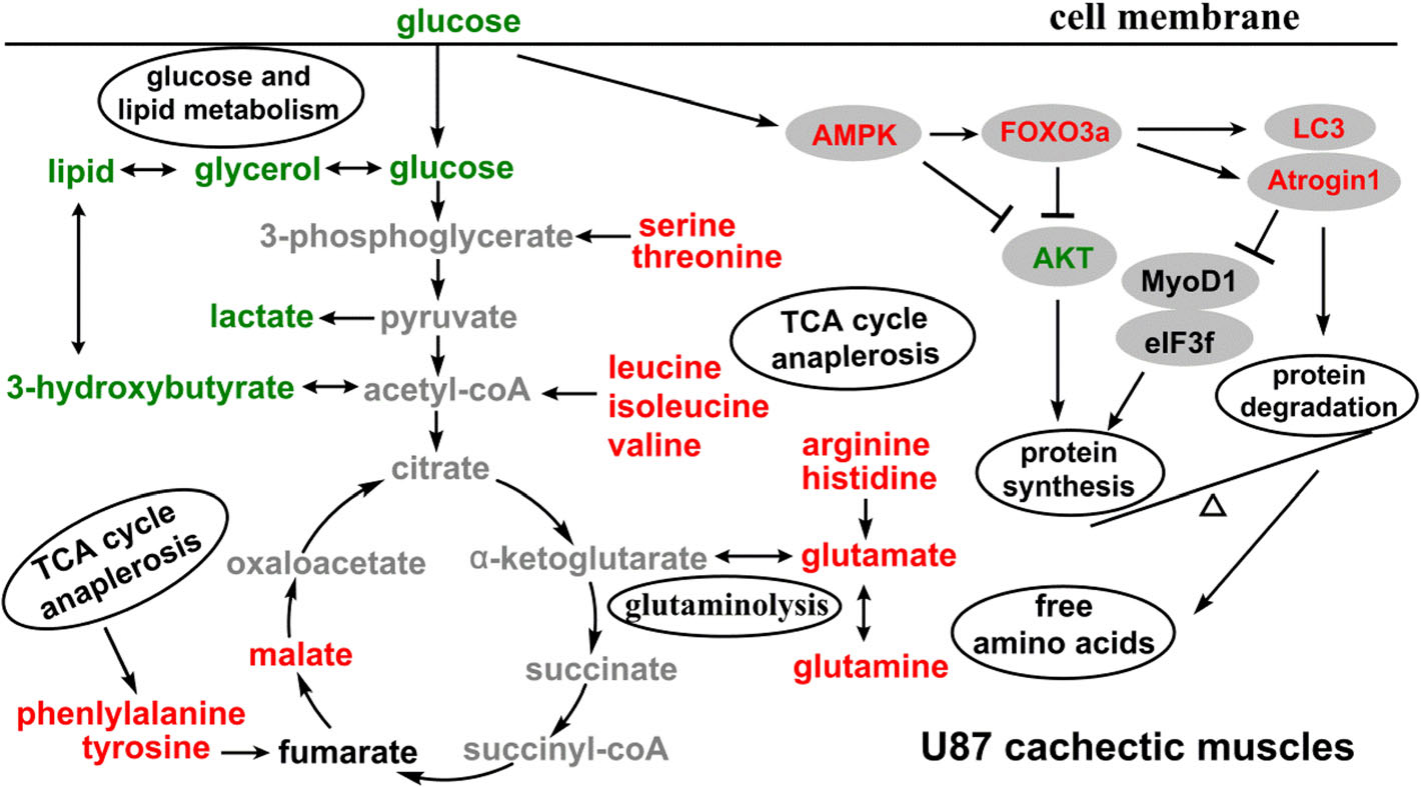
Overview of significantly altered signaling pathways and metabolic pathways involved in cachectic gastrocnemius of U87 mice relative to normal controls. Downregulated metabolites and proteins are colored green, whereas upregulated metabolites and proteins are colored red. Decreased metabolites as main energy sources include glucose, glycerol and 3-hydroxybutyrate, and amino acids largely released from AMPK-FOXO-Atrogin1/LC3-mediated proteolysis are mostly involved in glucose and lipid metabolism, and TCA cycle anaplerosis, including BCAAs, glutamine, glutamate, phenylalanine, tyrosine, histidine, arginine, serine, threonine, and TCA cycle intermediate malate. Furthermore, AKT-mediated protein synthesis is suppressed in U87 gastrocnemius

Skeletal muscle is a major site of metabolic activity and the most abundant tissue in human body accounting for almost 50% of total body mass [32]. Previous studies have shown metabolic disorders in cachectic muscles during cancer cachexia [16, 33]. Our metabolomic data reveal the distinct metabolic profiles of glioma-bearing mice were closely associated with the malignant grades of glioma. Compared with the low-grade CHG5 mice, the high-grade U87 mice displayed much more differential metabolites in gastrocnemius. Overall, the predominant metabolic changes of glioma cachexia involve glucose and lipid metabolism, protein catabolism and anabolism, and citric acid cycle (TCA) anaplerotic (Fig. 7).

Glucose and lipids are known as main energy sources in cellular metabolisms. Altered glucose and lipid metabolism in cancer cachexia is associated with muscle atrophy [34, 35]. Deficiency of glucose and depletion of glycogen store were observed in C26 cachectic muscles [33], and decreased levels of glucose and glycerol were detected in sera of C26 mice [20]. Furthermore, AMPK acts as a sensor of cellular energy that is typically activated in skeletal muscle during glucose deprivation [36]. Similarly, we observed significantly reduced energy sources (glucose, 3-HB) and activated AMPK in cachectic muscles in a malignant grade-dependent manner. We also observed distinctly decreased glucose level in the sera of U87 mice (Additional file 1: Table S3), indicative of hypoglycemia in line with the decreased glucose level in the cachectic muscle. Moreover, U87 mice showed significantly downregulated glycerol level in gastrocnemius, while CHG5 mice did not show statistically significantly changed glycerol level. As it is known, ketone bodies can substitute for glucose as substrates for the brain, muscle, and indeed most other tissues during severe energy deficiency [37]. As a major component of ketone bodies, 3-hydroxybutyrate was downregulated in CHG5 and U87 cachectic muscles, suggesting an increased usage of ketone bodies to substitute for exhaustion of glucose. During cancer cachexia, tumors may reprogram the skeletal muscle metabolism to meet a fast requirement of energy sources [16]. Overall, we suggest that glioma tumors usurped energy sources through highly activated glycolysis and lipolysis during glioma cachexia progression. These phenomena were more prominent in U87 mice than CHG5 mice.

As in existing cachexia studies, our data demonstrate that muscle catabolism is triggered primarily by upregulated E3 ligases, which is a pivotal aspect of skeletal muscle loss. Levels of Atrogin1 and MuRF1 are rapidly enhanced in numerous models of muscle atrophy, suggesting that both proteins might contribute to the initiation of an atrophy program [38]. Our data highlight the importance of Atrogin1 in glioma-induced muscle atrophy. Unexpectedly, we did not observe upregulated MuRF1. It might be one way in which glioma cachexia differs from other cancer cachexia. In addition, previous works showed that FOXO3 could upregulate Atrogin1, LC3, which was able to be activated by AMPK [39–41]. Our results also suggest that AMPK phosphorylation regulates Atrogin1 and LC3 expressions via the FOXO family and promotes protein breakdown.

As a result of promoted muscle proteolysis, free amino acids were largely released. A previous work observed reduced 3-carbon glycolytic and TCA intermediates in C26 cachectic muscles, which was defined as a Warburg-like effect [16]. Interestingly, we found decreased lactate and 11 increased amino acids in U87 cachectic muscles (isoleucine, leucine, valine, arginine, glutamate, glutamine, serine, threonine, tyrosine, histidine, and phenylalanine). The downregulation of lactate might arise from the exhaustion of glucose as a major substrate. As it is known, supplementation with branched-chain amino acids (BCAAs), especially leucine, could activate mTOR signaling and improve skeletal muscle metabolism [42, 43]. Moreover, glutamine could improve skeletal muscle cell differentiation and prevents myotubes atrophy via reduced p38 MAPK signal transduction [44]. Thus, BCAAs and glutamine might either act as precursors to promote protein synthesis, or be metabolized as replenish citric acid cycle intermediates.

In addition, when energy sources are deficient, AMPK usually acts as a metabolic checkpoint which could inhibit cellular growth involving glycogenesis, fatty acids synthesis, and protein biosynthesis [45]. Activation of AMPK induces the repressed protein synthesis by downregulating the mTOR signaling [46]. AKT pathways could promote muscle growth via mTOR and simultaneously block protein degradation by inhibiting FOXO [47, 48]. Our results suggest that downregulated AKT mediates protein biosynthesis in glioma cachectic muscles. On the other hand, as two most widely identified substrates of Atrogin1 in skeletal muscle, MyoD and eIF3f play vital roles in control of muscle protein synthesis [49]. However, our work observed unchanged levels of MyoD1 and eIF3f. Taken together, these results suggest that the repression of muscle protein synthesis is primarily through downregulating the AKT rather than the MyoD and eIF3f.

In accordance with the markedly altered muscle atrophy-related proteins, MSEA suggests that protein biosynthesis is the most significantly disturbed metabolism both in CHG5 and U87 mice relative to controls. Besides the in vivo experiments described above, we also conducted NMR-based metabolomic profiling of aqueous extracts derived from murine C2C12 myotubes after exposure to HEB, CHG5 and U87 conditioned media (CM). The PCA scores plot displays distinctly distinguished metabolic profiles of three groups of myotubes (data not shown). Moreover, MSEA shows that protein biosynthesis is the top-ranking significantly altered metabolism in the C2C12 myotubes after treatment with either CHG5 CM or U87 CM, relative to HEB CM (data not shown). Overall, these results indicate that significantly disturbed protein biosynthesis is associated with skeletal muscle atrophy. Therefore, it seems that these metabolites such as BCAAs and glutamine did not act as precursors to promote protein synthesis, but might be metabolized as replenish citric acid cycle intermediates for anaplerotic reactions and consequently oxidative phosphorylation.

BCAAs can be converted to acetyl-CoA and then enter the TCA cycle. Glutamine is involved in anaplerosis through glutaminolysis, which enters the TCA cycle by being converted into glutamate initially and then into α-ketoglutarate [9, 50]. Interestingly, U87 mice showed increased glutamate and glutamine, while CHG5 mice displayed slightly increased glutamate and non-significantly changed glutamine, implying the distinct rates of glutaminolysis and different utilizations of glutamine in CHG5 and U87 mice. Furthermore, serine and threonine can join in glycolysis by being converted into pyruvate from 3-phosphoglycerate [16, 51]. Additionally, arginine and histidine can be transformed into TCA intermediates α-ketoglutarate [51]. In our work, the 11 amino acids were upregulated in U87 gastrocnemius muscles, suggesting that more active TCA cycle anaplerotic flux might occur to meet the energy demands during severe muscle atrophy. These results are supported by both a higher uptake of glucose and the significant increase of the TCA intermediate malate. However, previous studies have indicated impaired oxidative phosphorylation system (OXPHOS) function/efficiency in cachectic gastrocnemius and specifically during muscle atrophy [52, 53]. It seems that even though elevating TCA cycle-enterable precursors may be a compensatory attempt for energy provision, it is ultimately not effective due to impaired mitochondrial OXPHOS. Overall, the largely increased amino acids might result from skeletal muscle proteolysis, failure of the normal stimuli for muscle protein synthesis, and impaired OXPHOS function.

To our knowledge, this work represents the first study to explore the malignant grade-dependent glioma cachexia and metabolic derangements of skeletal muscle in a glioma murine model. We have demonstrated that both the specific cachexia symptoms and metabolic profiles of glioma are closely associated with the malignant grades and expanded the mechanistic understanding of glioma-induced muscle wasting. Future investigations are essential to conduct metabolic profiling of sera occurring in glioma cachexia. Such investigations would be beneficial to comprehensively understand glioma-induced metabolic derangements and discover specific biomarkers as well as provide molecular basis for future treatments of gliomas.

## Conclusion

In summary, our findings provide significant insight into the complex pathophysiology of glioma cachexia correlated with malignant grades and highlight the importance of AMPK, FOXO, Atrogin1, LC3, and AKT in glioma-induced muscle atrophy. We also found that energy sources are remarkably deficient and amino acids are largely released in cachectic muscles. Significantly, high-grade glioma mice exhibit more severe cachexia symptoms and complex metabolic derangements than low-grade glioma mice. In addition, the metabolic changes identified here might provide new treatments to glioma patients. For example, our findings suggest that ketone bodies are supplementations preferred more than BCAAs for the treatment of catabolic states during glioma cachexia. Further therapeutic interventions on glioma patients can be designed to specifically downregulate or inhibit effector molecules like Atrogin1 and, specifically, upregulate and activate AKT, as well as efficiently implement nutritional supplementations such as ketogenic diets against glioma cachexia.

## Additional file

Additional file 1: **Table S1.** Relevant references for the used antibodies. **Figure S1.** Typical 2D ^1^H-^13^C HSQC spectrum of the aqueous extract derived from the gastrocnemius muscle of a HEB mouse. The spectrum was recorded at 25 °C on a Bruker Advance III 850 MHz NMR spectrometer. The inserted dashed box shows the amplification map of the region ^1^H (3.0–4.6 ppm) and ^13^C (60–80 ppm) of the full spectrum. The serial numbers indicate the following metabolites: 1, isoleucine; 2, valine; 3, leucine; 4, ethanol; 5, 3-hydroxybutyrate; 6 lactate; 7, alanine; 8, lysine; 9, glutamine; 10, glutathione; 11, glutamate; 12, creatine; 13, taurine; 14, inosinate; 15, glycine; 16, glycerol; 17, choline; 18, myoinositol; 19, glucose; 20, serine; 21, carnosine; 22, anserine; 23, fumarate; 24, phenylalanine; 25, niacinamide; 26, acetate; 27, tyrosine; 28, threonine; 29, aspartate; 30, asparagine; 31, malate; 32, inosine; 33, mannose; 34, histidine; 35, arginine; 36, phosphocholine; 37, NAD+; 38, glycerophosphocholine (GPC). **Figure S2.** PLS-DA score plots and validation plots of 1D ^1^H NMR data for aqueous extracts derived from gastrocnemius muscles of mice. (A), (D) CHG5 vs. HEB mice; (B), (E) U87 vs. HEB mice; (C), (F) U87 vs. CHG5 mice. The PLS-DA models were cross-validated to evaluate the robustness by a random permutation test (200 cycles). *n* = 6–7 mice/group. **Table S2.** Comparison of metabolite levels between the three groups of mice based on relative integrals calculated from the 1D ^1^H NMR spectra of aqueous gastrocnemius extracts. **Table S3.** Comparison of glucose levels between the three groups of mice based on relative integrals calculated from the 1D ^1^H NMR spectra of sera. (DOCX 439 kb)

## Abbreviations

3-HB: 3-Hydroxybutyrate
AMPK: AMP-activated protein kinase
Atrogin1/MAFbx: Muscle atrophy F-box
BCAAs: Branched-chain amino acids
CNS: Central nervous system
eIF3f: Eukaryotic initiation factor 3
FOXO: Forkhead box O
H&E: Hematoxylin and eosin
HMDB: Human Metabolome Data Base
HSQC: Heteronuclear single quantum coherence
LC3: Microtubule-associated protein 1 light chain 3
MSEA: Metabolite set enrichment analysis
MuRF1: Muscle ring finger 1
MyoD1: myogenic differentiation 1
NMR: Nuclear magnetic resonance
OXPHOS: Oxidative phosphorylation system
PCA: Principal component analysis
PLS-DA: Partial least-squares discriminant analysis
TCA cycle: Citric acid cycle
WHO: World health organization
