# Supplementary material for: Metabolic derangements of skeletal muscle from a murine model of glioma cachexia
Source: Skelet Muscle. 2019 Jan 11;9:3. doi: 10.1186/s13395-018-0188-4 (PMC6330447; doi:10.1186/s13395-018-0188-4)
Supplement: Supplementary file 1 — Table S1. Relevant references for the used antibodies. Figure S1. Typical 2D 1H-13C HSQC spectrum of the aqueous extract derived from the gastrocnemius muscle of a HEB mouse. The spectrum was recorded at 25 °C on a Bruker Advance III 850 MHz NMR spectrometer. The inserted dashed box shows the amplification map of the region 1H (3.0–4.6 ppm) and 13C (60–80 ppm) of the full spectrum. The serial numbers indicate the following metabolites: 1, isoleucine; 2, valine; 3, leucine; 4, ethanol; 5, 3-hydroxybutyrate; 6 lactate; 7, alanine; 8, lysine; 9, glutamine; 10, glutathione; 11, glutamate; 12, creatine; 13, taurine; 14, inosinate; 15, glycine; 16, glycerol; 17, choline; 18, myoinositol; 19, glucose; 20, serine; 21, carnosine; 22, anserine; 23, fumarate; 24, phenylalanine; 25, niacinamide; 26, acetate; 27, tyrosine; 28, threonine; 29, aspartate; 30, asparagine; 31, malate; 32, inosine; 33, mannose; 34, histidine; 35, arginine; 36, phosphocholine; 37, NAD+; 38, glycerophosphocholine (GPC). Figure S2. PLS-DA score plots and validation plots of 1D 1H NMR data for aqueous extracts derived from gastrocnemius muscles of mice. (A), (D) CHG5 vs. HEB mice; (B), (E) U87 vs. HEB mice; (C), (F) U87 vs. CHG5 mice. The PLS-DA models were cross-validated to evaluate the robustness by a random permutation test (200 cycles). n = 6–7 mice/group. Table S2. Comparison of metabolite levels between the three groups of mice based on relative integrals calculated from the 1D 1H NMR spectra of aqueous gastrocnemius extracts. Table S3. Comparison of glucose levels between the three groups of mice based on relative integrals calculated from the 1D 1H NMR spectra of sera. (DOCX 439 kb) [file 13395_2018_188_MOESM1_ESM.docx]

**Metabolic derangements of skeletal muscle from a murine model of glioma cachexia**

Pengfei Cui^1^, Wei Shao^2^, Caihua Huang^3*^, ChangJer Wu^4^, Bin Jiang^5^, Donghai Lin^1*^

^1^Department of Chemical Biology, College of Chemistry and Chemical Engineering, Xiamen University, Xiamen, China;

^2^Department of Pathology, Affiliated Chenggong Hospital of Xiamen University, Xiamen, China;

^3^Department of Physical Education, Xiamen University of Technology, Xiamen, China;

^4^Department of Food Science, National Taiwan Ocean University, Keelung, Taiwan;

^5^State Key Laboratory of Cellular Stress Biology, School of Life Sciences, Xiamen University, Xiamen, China.

*Correspondence authors: Donghai Lin, [dhlin@xmu.edu.cn](mailto:dhlin@xmu.edu.cn); Caihua Huang, [huangcaihua@xmut.edu.cn](mailto:huangcaihua@xmut.edu.cn).

Table S1. Relevant references for the used antibodies.

| **Antibodies** | **References** |
| --- | --- |
| p-AMPK (Thr172) (2535S; Cell Signaling Technology). | Metformin Suppresses Retinal Angiogenesis and Inflammation in Vitro and in Vivo. *PLoS One* 13, e0193031, 2018. |
| AMPK (10929; Proteintech). | AMPK Deficiency in Chondrocytes Accelerated The Progression of Instability-induced and Ageing-associated Osteoarthritis in Adult Mice. *Sci Rep* 7: 1-14, 2017. |
| p-AKT (Thr308) (13038S; Cell Signaling Technology). | Deletion of PDK1 Causes Cardiac Sodium Current Reduction in Mice. *Eur Heart J* 36: 1123-1123, 2015. |
| AKT (4685S; Cell Signaling Technology). | Muscle-specific Knock-out of NUAK Family SNF1-like Kinase 1 (NUAK1) Prevents High Fat Diet-induced Glucose Intolerance. *J Biol Chem* 287: 16379-16389, 2012. |
| p-AKT (Ser473) (9271S; Cell Signaling Technology).  p-FOXO3a (Ser253) (9466S; Cell Signaling Technology). | The Mitochondrial Metabolic Reprogramming Agent Trimetazidine as An 'Exercise Mimetic' in Cachectic C26-bearing Mice. *J Cachexia Sarcopenia Muscle* 8: 954-973, 2017. |
| FOXO3a (10849; Proteintech). | Rictor/mTORC2 Pathway in Oocytes Regulates Folliculogenesis, and Its Inactivation Causes Premature Ovarian Failure. *J Biol Chem* 290: 6387-6396, 2015. |
| LC3 (12135; Proteintech). | Autophagy is essential for mouse sense of balance. *J Clin Invest.* 120: 2331-2344, 2010. |
| MyoD1 (ab64159; Abcam). | Potential of Adipose-Derived Mesenchymal Stem Cells and Skeletal Muscle-Derived Satellite Cells for Somatic Cell Nuclear Transfer Mediated Transgenesis in Arbas Cashmere Goats. *PLoS One* 9, e93583, 2014. |
| MuRF1 (ab172479; Abcam).  Fbx32 (ab168372; Abcam). | Involvement of the FoxO1/MuRF1/Atrogin-1 Signaling Pathway in the Oxidative Stress-Induced Atrophy of Cultured Chronic Obstructive Pulmonary Disease Myotubes. *PLoS One* 11, e0160092, 2016. |
| eIF3f (ab176853; Abcam). | Dexamethasone Downregulates Caveolin-1 Causing Muscle Atrophy via Inhibited Insulin Signaling. *J Endocrinol* 225: 27-37, 2015. |
| GAPDH (10494; Proteintech). | The Expression of a Mitochondria-Localized Glutamic Acid-Rich Protein (MGARP/OSAP) Is Under the Regulation of the HPG Axis. *Endocrinology* 152: 2311-2320, 2011. |


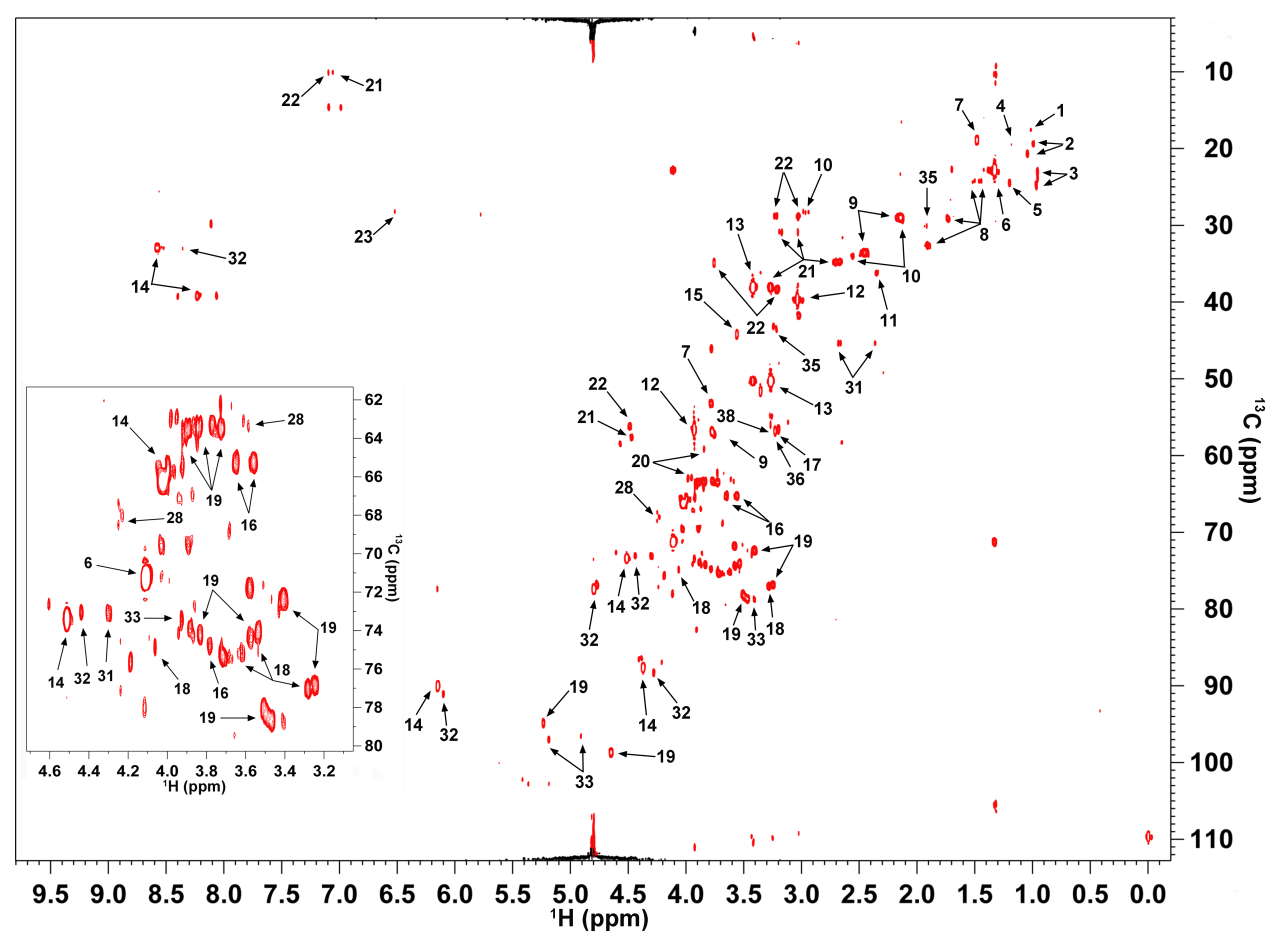


Fig S1. Typical 2D ^1^H-^13^C HSQC spectrum of the aqueous extract derived from the gastrocnemius muscle of a HEB mouse. The spectrum was recorded at 25 °C on a Bruker Advance III 850 MHz NMR spectrometer. The inserted dashed box shows the amplification map of the region ^1^H (3.0-4.6 ppm) and ^13^C (60-80 ppm) of the full spectrum. The serial numbers indicate the following metabolites: 1, isoleucine; 2, valine; 3, leucine; 4, ethanol; 5, 3-hydroxybutyrate; 6 lactate; 7, alanine; 8, lysine; 9, glutamine; 10, glutathione; 11, glutamate; 12, creatine; 13, taurine; 14, inosinate; 15, glycine; 16, glycerol; 17, choline; 18, myoinositol; 19, glucose; 20, serine; 21, carnosine; 22, anserine; 23, fumarate; 24, phenylalanine; 25, niacinamide; 26, acetate; 27, tyrosine; 28, threonine; 29, aspartate; 30, asparagine; 31, malate; 32, inosine; 33, mannose; 34, histidine; 35, arginine; 36, phosphocholine; 37, NAD+; 38, glycerophosphocholine (GPC).


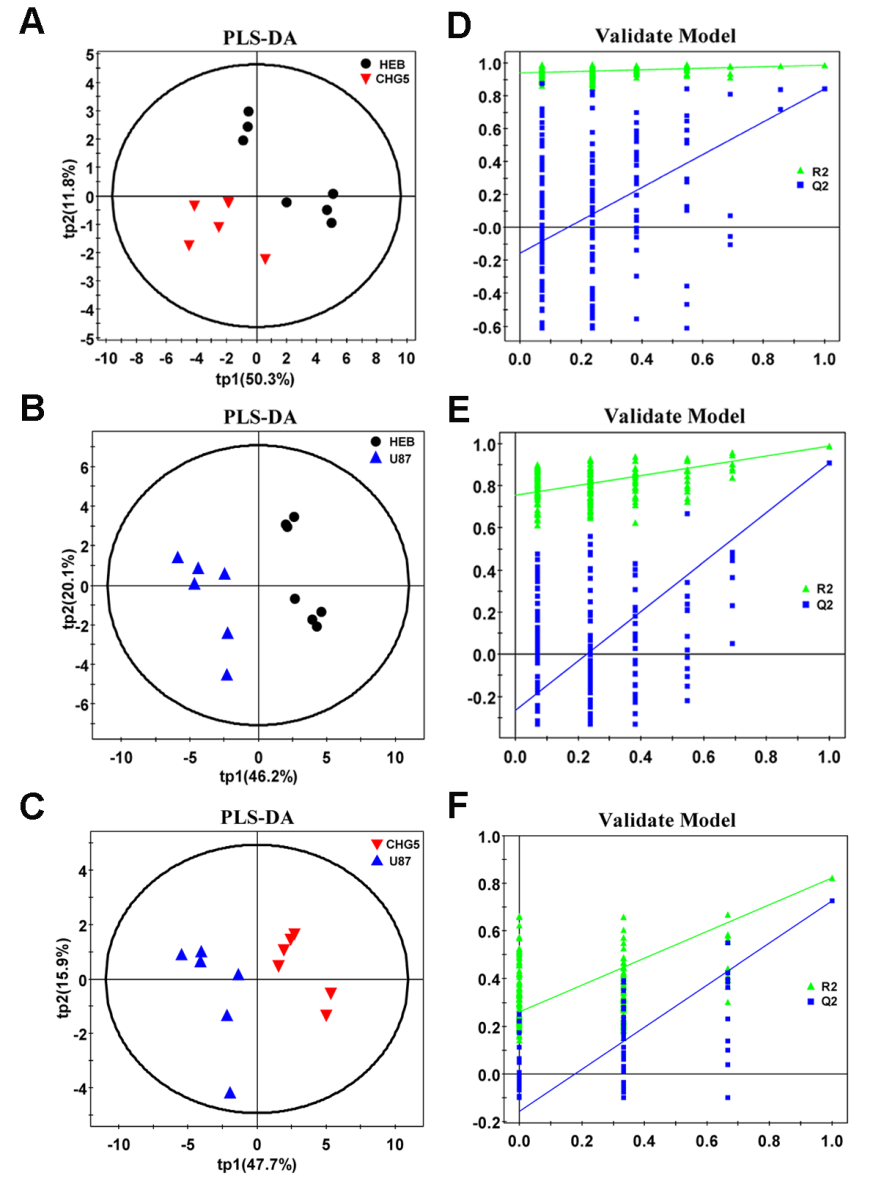


Fig S2. PLS-DA score plots and validation plots of 1D ^1^H NMR data for aqueous extracts derived from gastrocnemius muscles of mice. (A), (D) CHG5 vs. HEB mice; (B), (E) U87 vs. HEB mice; (C), (F) U87 vs. CHG5 mice. The PLS-DA models were cross-validated to evaluate the robustness by a random permutation test (200 cycles). n=6-7 mice/group.

Table S2. Comparison of metabolite levels between the three groups of mice based on relative integrals calculated from the 1D ^1^H NMR spectra of aqueous gastrocnemius extracts.

| Metabolites | Mean ± standard deviation | | | CHG5 | U87 | U87 |
| --- | --- | --- | --- | --- | --- | --- |
|  | HEB | CHG5 | U87 | versus  HEB | versus  HEB | versus CHG5 |
| isoleucine | 0.051±0.010 | 0.069±0.005 | 0.112±0.017 | * | *** | *** |
| leucine | 0.305±0.053 | 0.424±0.024 | 0.618±0.091 | ** | *** | *** |
| valine | 0.136±0.023 | 0.157±0.010 | 0.297±0.061 | NS | *** | *** |
| ethanol | 0.12±0.0470 | 0.085±0.027 | 0.082±0.012 | NS | NS | NS |
| 3-HB | 0.197±0.023 | 0.137±0.030 | 0.053±0.003 | *** | *** | *** |
| lactate | 18.182±1.066 | 17.784±1.739 | 8.638±3.816 | NS | *** | *** |
| alanine | 1.015±0.106 | 1.058±0.046 | 1.033±0.123 | NS | NS | NS |
| arginine | 0.161±0.017 | 0.200±0.013 | 0.241±0.023 | ** | *** | ** |
| lysine | 0.197±0.016 | 0.193±0.010 | 0.218±0.028 | NS | NS | NS |
| acetate | 0.149±0.015 | 0.137±0.012 | 0.160±0.027 | NS | NS | NS |
| glutamate | 0.207±0.031 | 0.314±0.089 | 0.343±0.052 | * | ** | NS |
| glutamine | 0.919±0.026 | 0.816±0.067 | 1.039±0.136 | NS | * | ** |
| glutathione | 0.314±0.024 | 0.286±0.018 | 0.338±0.030 | NS | NS | ** |
| aspartate | 0.032±0.007 | 0.041±0.011 | 0.032±0.004 | NS | NS | NS |
| asparagine | 0.021±0.008 | 0.030±0.020 | 0.024±0.010 | NS | NS | NS |
| creatine | 11.055±0.545 | 11.159±0.575 | 12.867±0.597 | NS | *** | NS |
| choline | 0.173±0.058 | 0.177±0.043 | 0.270±0.030 | NS | ** | ** |
| taurine | 9.404±0.389 | 9.523±0.366 | 11.462±0.524 | NS | *** | *** |
| glycine | 0.755±0.060 | 0.493±0.051 | 0.564±0.047 | *** | *** | NS |
| mannose | 0.015±0.001 | 0.014±0.004 | 0.017±0.002 | NS | NS | NS |
| glycerol | 0.529±0.066 | 0.461±0.062 | 0.349±0.015 | NS | *** | ** |
| anserine | 0.607±0.041 | 0.603±0.021 | 0.646±0.104 | NS | NS | NS |
| serine | 0.39±0.0380 | 0.31±0.1080 | 0.446±0.065 | NS | * | * |
| myoinositol | 0.138±0.015 | 0.129±0.025 | 0.244±0.020 | NS | *** | *** |
| threonine | 0.124±0.005 | 0.105±0.018 | 0.164±0.013 | NS | * | *** |
| malate | 0.039±0.003 | 0.041±0.005 | 0.047±0.004 | NS | ** | ** |
| inosinate | 0.774±0.036 | 0.802±0.033 | 0.828±0.039 | NS | * | NS |
| glucose | 0.205±0.044 | 0.139±0.005 | 0.075±0.031 | ** | *** | *** |
| inosine | 0.056±0.007 | 0.056±0.011 | 0.098±0.026 | NS | ** | ** |
| fumarate | 0.026±0.004 | 0.025±0.010 | 0.027±0.004 | NS | NS | NS |
| tyrosine | 0.034±0.004 | 0.036±0.007 | 0.053±0.010 | NS | *** | ** |
| carnosine | 0.143±0.014 | 0.117±0.057 | 0.162±0.005 | NS | ** | NS |
| histidine | 0.024±0.002 | 0.021±0.005 | 0.029±0.001 | NS | *** | ** |
| phenylalanine | 0.052±0.007 | 0.056±0.013 | 0.095±0.010 | NS | *** | *** |
| niacinamide | 0.057±0.003 | 0.051±0.008 | 0.065±0.002 | NS | * | ** |
| NAD+ | 0.009±0.001 | 0.008±0.002 | 0.014±0.001 | NS | *** | *** |
| PC | 0.419±0.102 | 0.437±0.103 | 1.043±0.070 | NS | *** | *** |
| GPC | 1.024±0.061 | 0.947±0.046 | 1.136±0.121 | NS | NS | ** |

Note: NS (P > 0.05), *P < 0.05, **P < 0.01 and ***P < 0.001 for CHG5 or U87 vs. HEB, and U87 vs. CHG5 as determined by one-way ANOVA. Red and blue colors denote that the difference is positive (i.e. A was increased compared to B) and negative, respectively. 3-HB, 3-hydroxybutyrate; PC, phosphocholine; GPC, glycerophosphocholine.

Table S3. Comparison of glucose levels between the three groups of mice based on relative integrals calculated from the 1D ^1^H NMR spectra of sera.

| Metabolites | Mean ± standard deviation | | | CHG5 | U87 | U87 |
| --- | --- | --- | --- | --- | --- | --- |
|  | HEB | CHG5 | U87 | versus  HEB | versus HEB | versus CHG5 |
| glucose | 2.187±0.228 | 2.503±0.267 | 1.258±0.273 | NS | *** | *** |

Note: NS (P > 0.05), ***P < 0.001 for U87 vs. HEB or CHG5 as determined by one-way ANOVA. Blue colors denote that the difference is negative.
